# Factors related to Gaming addiction in adults

**DOI:** 10.1192/j.eurpsy.2022.934

**Published:** 2022-09-01

**Authors:** C. Neily, M. Maalej, I. Gassara, R. Feki, N. Smaoui, L. Zouari, A. Zouari, J. Ben Thabet, S. Omri, N. Charfi, M. Maalej

**Affiliations:** Hedi Chaker hospital, Psychiatry Department, Sfax, Tunisia

**Keywords:** gaming, Addiction, adults

## Abstract

**Introduction:**

With the advancement of technology over the last years, gaming is no longer reserved to adolescents. It has become a growing phenomenon within young adults which should,’t be overlooked as it is accompanied with the risk of addiction.

**Objectives:**

To study the factors involved in video games addiction behaviors in adults

**Methods:**

We conducted a cross-sectional, descriptive and analytical study. Data were collected using a self-administered questionnaire on social networks. We solicited adults between 18–40 years. We used the gaming addiction scale (GAS) in its validated Arabic short version.

**Results:**

A hundred and nine participants were included. The mean age was 29.6 ±10.3 with a sex ratio of 1.5.The mean age of the beginning of regular gaming was 16.3± 8.64. we found that40.4% of our participants preferred the mode Massively Multiplayer Online Role Playing Games (MMORPG) while others played casual single player games. A play time of over 20 hours per week was reported by 11.9%of participants. According to the GAS, 25.7%were addicted gamers. Our participants spent an average of 7.94±6.71 hours before they play their first game of the day. We found that the score of Gas was significantly correlated to the male gender of the participants (p<0.000), a higher number of weekly gaming hours (p<0.000),a lower number of hours before gaming (P<0.000) and the mode of games (p<0.000).

**Conclusions:**

Our study showed that contextual factors play an important role in understanding gaming addiction in young adults as a holistic phenomenon,embedding the problematic behavior within the context of the individual the game and gaming practices.

**Disclosure:**

No significant relationships.

